# Neuro-Immune Aspects of Schizophrenia with Severe Negative Symptoms: New Diagnostic Markers of Disease Phenotype

**DOI:** 10.17691/stm2021.13.6.03

**Published:** 2021-12-28

**Authors:** I.K. Malashenkova, V.L. Ushakov, N.V. Zakharova, S.A. Krynskiy, D.P. Ogurtsov, N.A. Hailov, E.I. Chekulaeva, A.Y. Ratushnyy, S.I. Kartashov, G.P. Kostyuk, N.A. Didkovsky

**Affiliations:** Head of the Laboratory of Molecular Immunology and Virology; National Research Center “Kurchatov Institute”, 1 Akademika Kurchatova Square, Moscow, 123182, Russia; Leading Researcher, Laboratory of Clinical Immunology; Federal Research and Clinical Center of Physical-Chemical Medicine,; Associate Professor, Senior Researcher; National Research Nuclear University MEPhI, 31 Kashirskoe Shosse, Moscow, 115409, Russia; Department Head; Alekseev Psychiatric Clinical Hospital No.1, Moscow Department of Health, 2 Zagorodnoe Shosse, Moscow, 117152, Russia; Leading Researcher, Institute for Advanced Brain Research; Lomonosov Moscow State University, 27/1 Lomonosov Avenue, Moscow, 119192, Russia;; Head of the Laboratory for Fundamental Research Methods; Alekseev Psychiatric Clinical Hospital No.1, Moscow Department of Health, 2 Zagorodnoe Shosse, Moscow, 117152, Russia;; Researcher, Laboratory of Molecular Immunology and Virology; National Research Center “Kurchatov Institute”, 1 Akademika Kurchatova Square, Moscow, 123182, Russia;; Researcher, Laboratory of Molecular Immunology and Virology; National Research Center “Kurchatov Institute”, 1 Akademika Kurchatova Square, Moscow, 123182, Russia; Researcher, Laboratory of Clinical Immunology; Federal Research and Clinical Center of Physical-Chemical Medicine,; Senior Researcher, Resource Center for Molecular and Cellular Biology; National Research Center “Kurchatov Institute”, 1 Akademika Kurchatova Square, Moscow, 123182, Russia;; Junior Researcher, Resource Center for Molecular and Cellular Biology; National Research Center “Kurchatov Institute”, 1 Akademika Kurchatova Square, Moscow, 123182, Russia;; Researcher, Laboratory of Cell Physiology; Russian Federation State Research Center Institute of Biomedical Problems of the Russian Academy of Sciences, 76A Khoroshevskoe Shosse, Moscow, 123007, Russia; Laboratory Deputy Head; National Research Center “Kurchatov Institute”, 1 Akademika Kurchatova Square, Moscow, 123182, Russia;; Professor, Chief Physician; Alekseev Psychiatric Clinical Hospital No.1, Moscow Department of Health, 2 Zagorodnoe Shosse, Moscow, 117152, Russia;; Professor, Head of the Laboratory of Clinical Immunology; Federal Research and Clinical Center of Physical-Chemical Medicine,

**Keywords:** schizophrenia with negative symptoms, paranoid schizophrenia, neurocognitive markers, neurobiological markers, interleukins

## Abstract

**The aim of the study** was to analyze the immune-inflammatory profile of patients with paranoid schizophrenia and relate it to the severity of negative symptoms and the MRI data in order to identify biomarkers of schizophrenia severity, search for new approaches to therapy, and control its effectiveness.

**Materials and Methods:**

The main group included 51 patients with paranoid schizophrenia, the control group — 30 healthy subjects. Patients underwent MRI scans and immunological studies, which included an assessment of natural and adaptive immunity, the systemic level of key pro-inflammatory and anti-inflammatory cytokines, and other markers of inflammation.

**Results:**

Disorders of immunity and immunoinflammatory profile in patients with paranoid schizophrenia with severe negative symptoms were revealed for the first time: in the presence of severe negative symptoms (>15 points according to the NSA-4 scale), the levels of humoral immunity factors, cytokines IL-10 and IL-12p40 and neurotrophin NGF were increased as well as the markers of systemic inflammation. Morphometric changes in the brain, typical for patients with schizophrenia, and also specific for patients with severe negative symptoms, were determined. The data analysis revealed correlations between the immune changes with structural changes in some of the brain areas, including the frontal cortex and hippocampus. Associations were found between the levels of anti-inflammatory IL-10, IL-12p40 cytokines and morphometric parameters of the brain, specific only for schizophrenic patients with severe negative symptoms.

**Conclusion:**

The interdisciplinary approach, combining brain morphometry with in-depth immunological and clinical studies, made it possible to determine neurobiological, immune, and neurocognitive markers of paranoid schizophrenia with severe negative symptoms. The results are important for further deciphering the pathogenesis of schizophrenia and its subtypes, as well as for the search for new approaches to the treatment of severe forms of the disease.

## Introduction

Schizophrenia is a chronic mental disorder that is caused by a complex palette of genetic, epigenetic, and detrimental environmental factors [1]. The disease is characterized by an erroneous perception of the surrounding reality and an impaired relationship with reality. Symptoms of schizophrenia include positive (delusions, hallucinations) and negative symptoms (social isolation, apathy, motivational deficits, poor speech, decreased emotional reactivity, and abulia), as well as cognitive changes, disorganized thinking, and psychomotor disorders [2]. It should be noted that schizophrenia leads not only to early disability (the peak incidence falls into 18–25 years of age) [2] but also to a decrease in life expectancy [3]. Due to health problems and a higher suicide rate, life expectancy in schizophrenia is about 10–25 years less than that in the general population [4]. Treatment resistance is observed in 30% or more of patients with schizophrenia, which further increases the severe socioeconomic burden of the disease [5].

The pathogenesis of the disease and its various forms has not been fully deciphered. It is assumed that endogenous and exogenous (infectious, toxic, social, and other) pathological stimuli interact with genetic and epigenetic factors both in the prenatal and postnatal periods, which leads to disruption of brain morphogenesis, central and peripheral immune activation, and the development of systemic inflammation [1, 6, 7]. Of great interest are data showing the critical role of immune disorders and neuro-inflammation in the pathogenesis of schizophrenia [6, 8, 9].

By now, more and more evidence has accumulated on interconnection between the immune system, the peripheral nervous system, and the brain. Virtually all neurotransmitters, including acetylcholine and dopamine, are secreted by circulating lymphocytes, and their receptors are expressed by these cells and play an important role in the immune-regulation and neuro-immune interactions. It is believed that macrophages and T cells are special points of cholinergic signal transmission through the parasympathetic nervous system from the brain to the spleen and vice versa [4]. Thus, a large amount of data has been accumulated showing that immune factors play an important role in the functions of a healthy brain [10, 11]. At the same time, Bennett and Molofsky [12] emphasize that, despite the correlations (identified by many authors) between indicators of immune function and mental illness, their significance remains largely unknown.

We previously reported on the presence of systemic inflammation in patients with paranoid schizophrenia, which was most pronounced in patients with the first episode of psychosis [13]. In general, it was found that along with the development of schizophrenia, a number of immune factors increased as well. Among them, there are acute-phase proteins, anti-inflammatory proteins, macrophage and lymphocyte pro-inflammatory factors, including cytokines and chemokines of cells of natural and adaptive immunity. These deviations from the normal status can be combined in various ways to create numerous immune patterns affecting the signaling and metabolic pathways in the periphery and in the brain [14]. On this general background, the immune profiles of patients with schizophrenia with severe negative symptoms have not been studied enough.

Negative symptoms are characteristic of schizophrenia and they are present in more than half of patients (from 60 to 90% according to the literature) [15]. A subtype of the disease called deficit schizophrenia has been described, which is characterized by primary and persistent negative symptoms and occurs in about 20% of patients [16]. These symptoms are common in people with treatment-resistant schizophrenia; moreover, these symptoms are believed to determine the severity of the disease and the development of cognitive deficits in these patients. It is important to note that the negative symptoms in schizophrenic patients, to a greater extent than the positive ones, are associated with functional impairment and poor prognosis. Modern antipsychotics do not provide adequate therapy for negative symptoms, which remains a serious problem in the treatment and management of patients with schizophrenia [16, 17].

Neuroimaging contributes a lot to the identification of clinically significant biological processes in schizophrenia and provides the opportunity to study specifics of the brain pathology *in vivo*, including structural brain abnormalities, impaired functional connectivity, and neurotransmitter systems disorders [18]. In many patients with schizophrenia, there are structural, functional, and metabolic disorders in the frontal lobe, yet the mechanisms underlying these changes are not yet fully understood [18–20].

Until now, there are no commonly accepted approaches to the use of neuroimaging data for diagnosing schizophrenia, predicting psychosis, selecting the therapy, and disease monitoring. One of the important ways of translating MRI data into clinical practice is the monitoring of neuro-immune changes and markers of systemic inflammation in patients with schizophrenia, combined with an analysis of the clinical picture, the disease course, and the prevailing symptomatology.

**The aim of the study** was to analyze the immune-inflammatory profile of patients with paranoid schizophrenia and relate it to the severity of negative symptoms and the MRI data in order to identify biomarkers of schizophrenia severity, search for new approaches to therapy, and control its effectiveness.

## Materials and Methods

The study included 51 patients (32 men and 19 women) with paranoid schizophrenia aged 18 to 40 years (the mean age was 28.5±6.2 years). The patients were hospitalized for treatment at the Alekseev Psychiatric Clinical Hospital No.1 (Moscow, Russia) in 2017–2019. At the time of admission, they presented with hallucinations and delusions as well as delusions of control, mental automatism, and verbal pseudo-hallucinations. The clinical picture fits the first rank symptoms according to the Kurt Schneider’s criteria and the definitions of schizophrenia by ICD-10 and DSM-5. In two-thirds of the patients, hallucinatory-delusional syndrome was combined with negative symptoms. There were no pronounced symptoms of depression in the examined patients.

In this study, we used the following exclusion criteria: the presence of manifest symptoms of schizophrenia at the time of examination, organic diseases of the central nervous system, somatic conditions in the stage of decompensation, acute or exacerbated infections, autoimmune, auto-inflammatory diseases, and/or substance abuse.

The patients were divided into two groups according to the severity of their negative symptoms ranked by the Negative Symptom Assessment (NSA-4) scale: group 1 (n=36) — a score of 15 or higher and group 2 (n=15) — with scores <15. Socio-demographic and clinical characteristics of the subjects are presented in Tables 1 and 2. Of all patients with schizophrenia, 25 patients with various NSA-4 scores underwent a dynamic study. The control group included 30 healthy subjects from the hospital staff, comparable in age and sex, without signs of mental disorders and not related to patients with schizophrenia or other mental illnesses.

**Table 1:** Socio-demographic characteristics of patients

Parameters	Group 1 (n=36) NSA-4≥15	Group 2 (n=15) NSA-4<15	Control group (n=30)
Age at the time of examination (years), M±σ	28.2±6.7	27.3±5.4	27.1±1.6
Gender:			
male	23	8	13
female	13	7	17
Family status:			
married	6	5	13
divorced	—	—	—
never married	25	10	17
Education:			
incomplete secondary	1	—	—
secondary	7	1	—
specialized secondary	8	1	—
incomplete higher education	8	4	4
higher education	12	9	26
Work:			
student	7	2	9
works	6	8	19
does not work	21	5	—
disabled person	2	—	2
Family history:			
aggravated	22	8	3
non-aggravated	9	5	25
not rated	5	2	2
Smoking:			
smokes	11	6	8
never smoked	12	9	21
quit over 2 years ago	5	—	1

**Table 2:** Clinical characteristics of patients, M±σ

Parameters	Group 1 (n=36) NSA-4≥15	Group 2 (n=15) NSA-4<15	p
Age at onset of prodromal symptoms, initial stage (years)	18.2±6.4	19.9±6.5	>0.05
Age at first manifest attack (years)	23.0±7.8	25.4±6.2	>0.05
Age at first seeking medical assistance (years)	21.4±6.5	25.1±5.9	>0.05
Age at first hospitalization in a psychiatric hospital (years)	23.0±7.5	25.1±5.9	>0.05
Duration of illness from the onset of prodromal symptoms (years)	5.9±6.5	2.4±4.0	**0.0268**
PANSS, total scores	102.5±20.2	76.7±28.9	**0.004**
PANSS (negative)	29.0±7.5	17.2±7.2	**0.00001**
BFCRS	6.4±8.5	5.7±7.2	>0.05
NSA-4	21.7±3.8	9.7±3.0	**<0.00001**
FAB	14.9±2.6	15.8±2.2	>0.05

For psychometric and neuropsychological examinations, the following tests and scales were used: the Positive and Negative Syndrome Scale (PANSS), the Bush–Francis Catatonia Rating Scale (BFCRS), the negative NSA-4 symptoms scale, and the Frontal Assessment Battery (FAB) test.

Immunological studies and MRI scans were carried out during the early stages of remission with a significant reduction in positive symptoms and the formation of criticism of the past psychosis. A decrease in positive symptoms occurred after standard therapy with atypical antipsychotics (olanzapine, risperidone) at the doses equivalent to 6–8 mg risperidone per day. In all patients, the following parameters were measured: levels of cytokines (IL-1β, TNF-α, IL-6, IL-2, IL-4, IL-8, IL-10, IFN-γ, and IL-12p40), C-reactive protein (CRP), cortisol, circulating immune complexes (CIC), total immunoglobulins (IgA, IgM, IgG), and neurotrophin NGF in the blood serum. The measurements were made using ELISA assays and reagent kits produced by Cytokine (Russia), Vector-Best (Russia), BioVendor — Laboratorni medicina a.s. (Czech Republic), Bender MedSystems (Austria), and XEMA (Russia).

MRI scanning was performed in 39 patients at the National Research Center “Kurchatov Institute” (Moscow, Russia) using a Magnetom Verio 3T magnetic resonance tomograph (Siemens, Germany). The images were obtained with the help of a 32-channel coil designed for brain imaging. In order to assess the gyrification, perform morphometry of the gray and white matter, and determine the volume of cerebrospinal fluid, anatomical data were obtained for each subject based on the T1-weighted sequence (TR=1900 ms; TE=2.21 ms; 176 slices; voxel size — 1×1×1 mm).

All the structural images were analyzed by the supercomputer of the National Research Center “Kurchatov Institute” using the Freesurfer program. It is an open-source software package that allows for assessing the subcortical and cortical segmentation, reconstructing the cortical surface, measuring the cortical thickness, and performing full brain morphometry without interference from a cranial signal. Based on the Freesurfer output data, the index of local cerebral gyrification was calculated.

### Ethical principles

The study was approved by the Ethics Committee of the National Research Center “Kurchatov Institute”. All participants were informed on the study details and signed a voluntary informed consent form, as well as consent to the processing of personal data. The principles of the Helsinki Declaration (2013) were strictly followed.

### Statistical processing

For statistical processing, the Excel (Microsoft, 2010) and Statistica 10.0 (StatSoft, 2010) software were used. The data distribution normality was assessed using the Shapiro–Wilk test. The group-specific results for clinical parameters were presented as means ± standard deviation (M±σ), for immunological parameters — as medians with 25^th^ and 75^th^ percentiles. To assess the significance of differences for clinical parameters, the Student’s t-test was used, for immunological parameters — the Mann–Whitney U-test, considering the differences between the parameters to be statistically significant at p<0.05. To assess the correlations, the Pearson correlation coefficient was used. The Chaddock scale was used to assess the strength of the identified correlations.

## Results

The study revealed a relationship between the presence of immune system mediators and the severity of negative and positive symptoms in paranoid schizophrenia. Thus, in patients with severe negative symptoms (group 1), the markers of humoral immunity activation were present at increased levels. Thus, the data for CIC were: 95.5 [68.0; 140.0] a.u. in group 1, 73 [58; 104] a.u. in group 2, and 83 [55; 99] a.u. in the control group (Figure (a)). In addition, there was a trend towards a higher level of class G immunoglobulins (13.53 [12.10; 15.42] g/L in group 1, 11.84 [10.80; 13.86] g/L in group 2, and 13.04 [10.16; 14.50] g/L — in control). Additionally, in some patients of group 1 with a pronounced increase in the CIC (18 out of 36 people, the median CIC level was 138.5 [118.0; 215.0] a.u.), an increase in IL-4, CRP, and IFN-γ was found, which indicated an activation of the adaptive immunity. Furthermore, these patients had a higher neutrophil-lymphocyte index (1.96 [1.67; 2.77] compared to 1.53 [1.13; 2.59] in group 2), which reflected a greater activation of their peripheral blood neutrophils/phagocytes.

**Figure:**
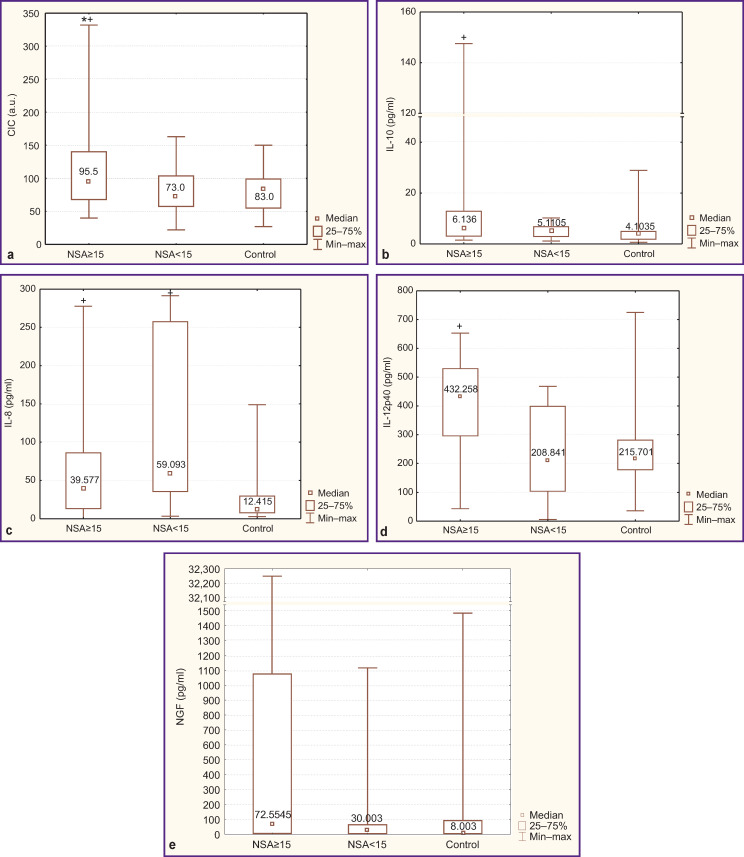
Serum levels of CIC, cytokines, and neurotrophin NGF in patients with paranoid schizophrenia with NSA-4 scores ≥15 (n=36), NSA-4 scores <15 (n=15), and in the control group (n=30): (a) CIC; (b) IL-10; (c) IL-8; (d) IL-12p40; (e) NGF; * statistically significant differences in values vs control; **^+^** between the groups of schizophrenic patients

In patients of group 1, the levels of IL-10 (Figure (b)) and IL-12p40 (Figure (d)), the anti-inflammatory mediators, were significantly higher than those in control and exceeded the respective values in patients of group 2. It turned out that in patients of group 1 with an increased presence of IL-10 (>5.4 pg/ml and a median of 7.73 [6.14; 24.62] pg/ml), a notable increase in the levels of CRP, CIC, cortisol, natural and adaptive immunity cytokines (IL-2, IL-4, IFN-γ) was detected more often. However, more than 40% of patients in group 1 with an increased level of CRP showed no respective increase in IL-10, NGF, and/or IL-12p40. At the same time, the level of IL-8, one of the main mediators of systemic inflammation, was higher than normal in patients with schizophrenia, regardless of the severity of their negative symptoms (Figure (c)).

In the majority of patients with severe negative symptoms, the serum neurotrophin NGF level was several-fold higher than that in the control group and that in patients with NSA-4 scores <15 (Figure (e)); however, the differences between the groups did not reach statistical significance due to large data variations.

In patients of group 1, a significant positive correlation was found between the levels of CRP, IL-4, IFN-γ, NGF, and the level of IL-10 (Table 3).

**Table 3:** Correlation of IL-10 level with the level of other immunological parameters in patients with schizophrenia

Parameters	Correlation coefficient	
	Group 1 (n=36) NSA-4≥15	Group 2 (n=15) NSA-4<15
CRP (mg/L)	0.79	0.13
IL-4 (pg/ml)	0.72	0.32
IFN-γ (pg/ml)	0.97	–0.42
NGF (pg/ml)	0.99	–0.03

The analysis of structural neuroimaging revealed a decrease (p<0.005) in the mean cortical thickness in several brain regions (mainly in the frontal lobes) in patients with schizophrenia, regardless of the severity of their negative symptoms. The affected areas included the right upper frontal gyrus, the opercular part of the left inferior frontal gyrus, the entire left hemisphere, the left postcentral gyrus, the rostral part of the left middle frontal gyrus, etc. In some areas, the mean cortical thickness decreased statistically significantly (p<0.005) only in patients with highly severe negative symptoms (NSA-4≥15): in the entire right hemisphere, in the triangular part of the right inferior frontal gyrus, in the caudal part of the left middle frontal gyrus, in the caudal part of the right middle frontal gyrus, in the triangular part of the left inferior frontal gyrus, in the rostral part of the right middle frontal gyrus, in the orbital part of the right inferior frontal gyrus, in the right superior temporal gyrus, etc. Notably, the mean thickness of the cortex in the right medial orbitofrontal region decreased significantly (p<0.005) only in patients with paranoid schizophrenia with less severe negative symptoms (NSA-4<15).

Taken together, these results (obtained for the first time) indicate that the cortex thickness in several zones of both hemispheres has changed; and these changes are characteristic only for patients with severe negative symptoms.

In the next stage, we looked into systemic immune-inflammatory changes associated with structural (morphometric) changes in the brain, especially in schizophrenic patients with severe negative symptoms. Specifically, we analyzed the relations between the levels of anti-inflammatory mediators and structural changes in the brain in schizophrenia, which are yet to be studied.

In patients with severe negative symptoms (NSA-4 score ≥15), the level of the anti-inflammatory cytokine IL-10 negatively correlated with the signal intensity in the basal nuclei and hippocampus (Table 4). Note that the similar correlations for the basal nuclei were also detected with the level of IL-12p40, which could indicate the existence of a specific disease profile associated with brain morphometric changes and with an increase in serum cytokines associated with the systemic inflammation.

**Table 4:** Parameters of structural MRI associated with IL-10 and IL-12p40 levels in schizophrenic patients with NSA-4 score ≥15

Parameters	Brain area	Correlation coefficient
***Associated with IL-10 level***		
Signal amplitude	Left caudate nucleus	–0.74
	Left nucleus accumbens	–0.58
	Right hippocampus	–0.49
	Left shell	–0.71
	Right caudate nucleus	–0.69
	Left hippocampus	–0.45
	Right nucleus accumbens	–0.68
	Right pallidus	–0.69
	Right shell	–0.72
	Left thalamus	–0.42
	Left pallidus	–0.67
***Associated with IL-12p40 level***		
Average curvature	Right transverse temporal gyrus	0.50
	Right superior temporal gyrus	0.50
Folding index	Pole of the right frontal lobe	0.85
	Left supramarginal gyrus	0.41
	Right calcarine sulcus	0.66
	Left calcarine sulcus	0.62
	Right superior temporal gyrus	0.61
	Right posterior cingulate gyrus	0.59
	Right lateral occipital cortex	0.45
Cortex area	Pole of the right temporal lobe	0.60
	Right calcarine sulcus	0.49
	Left calcarine sulcus	0.44
	Left transverse temporal gyrus	0.43
	Right posterior cingulate gyrus	0.64
	Isthmus of the left cingulate gyrus	0.42
	Right lateral occipital cortex	0.46
Average cortex volume	Right posterior cingulate gyrus	0.60
	Left tonsil	0.57
	Right calcarine sulcus	0.47

A number of associations specific only for schizophrenic patients with severe negative symptoms were identified between the level of the cytokine IL-12p40 and morphometric parameters. Thus, patients with an NSA-4 score ≥15 showed a significant (p<0.05) correlation between the IL-12p40 level and several parameters of structural MRI. Part of the data obtained is presented in Table 4.

## Discussion

In this study, for the first time, significant abnormalities in the immuno-inflammatory profile were revealed in schizophrenic patients with severe negative symptoms: an activation of the humoral immunity and a significant increase in the CIC level against the background of persisting low-level systemic inflammation. The data suggest that these patients are exposed to antigenic stimulation (endotoxins or other infectious antigens, brain tissue antigens, or other autoantigens). Endotoxins of intestinal origin are considered important because changes in the intestinal microbiota were shown to directly correlate with affective states, anxiety, and depression [21]. Also, in patients with schizophrenia, the clearance of immune complexes by phagocytes may be reduced, despite signs of phagocytic activation (the neutrophil-lymphocyte index and the level of cytokines of natural immunity are increased). In schizophrenic patients with severe negative symptoms, a decrease in the phagocytic activity and an increase in the cytokine production cannot be ruled out. In schizophrenia, microglia cells of the brain have a proinflammatory M1 phenotype and a reduced activity of phagocytosis [22–24]. The relationship between the state of microglia and peripheral phagocytes has also been suggested [9].

Patients with severe negative symptoms were found to have increased levels of the cytokines IL-10, IL-12p40, and NGF, known for their anti-inflammatory effects. Moreover, a significant positive correlation was found between the level of IL-10 and the levels of CRP, IL-4, IFN-γ, and NGF. At high levels of IL-10, we often observed a significant increase in the levels of CIC, cortisol, and cytokines of the natural and adaptive immunity (IL-2, IL-4, IFN-γ), which allowed us to characterize the elevated levels of IL-10 as a response to systemic inflammation. The key anti-inflammatory factor IL-10 is critical in protecting the body against tissue damage, especially during the acute phase of the immune response. The data indicate, on the one hand, a preserved immune response, and on the other hand, its insufficiency, since in patients with a high level of inflammation, the increased level of IL-10 does not prevent symptoms of inflammation. This inconsistence may be due to changes in the sensitivity of receptors to high levels of cytokines, insufficient activity of other anti-inflammatory factors, or the presence of polymorphisms in the IL-10 gene — all these mechanisms are possible and need further research. Importantly, high concentrations of IL-10 can be used by viruses to evade an immune attack. In particular, this is typical for herpes viruses, the activation of which can contribute to inflammation and, later, to neurodegeneration [25]. An excess of IL-10 may have its own negative effects: for example, in [26], it was shown that increased levels of peripheral IL-10 were associated with a disarrangement of the white matter microstructure in patients with schizophrenia.

In the present study, it has been found for the first time that in schizophrenic patients with severe negative symptoms, the level of IL-12p40 is significantly increased. Earlier in work [27], increased levels of IL-12p40 were found in schizophrenic patients, although the authors did not analyze the relationship between this change and the clinical symptoms or other parameters, except for gender. The 12p40 monomer inhibits the activity of IL-12 and production of IFN-γ, i.e. it has an anti-inflammatory effect on the activity of type 1 T helpers. There is evidence that IL-12p40 activates cells involved in anti-inflammatory effects and deactivates the autoimmune subpopulations of Th1 and Th17 helpers through suppression of IL-12 and IL-23 (by acting on their receptors and inducing IL-12Rβ1 internalization) [28]. The present results indicate that another anti-inflammatory mediator, IL-12p40, can suppress the antiviral response and contribute to chronic viral infections in schizophrenia.

As our studies have shown, the serum neurotrophin NGF level in patients with the most pronounced negative symptoms is several-fold higher than that in the control group and the group of patients without pronounced negative symptoms. NGF is involved in the development of neurons, their differentiation, maintenance, and growth during the life span. This neurotrophin has an impact on the structure and function of the brain, inducing the growth of neurites, their branching, and length [29, 30]. In the brain, the highest expression of NGF mRNA was found in the hippocampus [31]. It was found that the serum NGF level reflected its concentration in the central nervous system [32]. Animal studies have shown that NGF crosses the blood-brain barrier [33]. Moreover, several studies have reported an association between the peripheral NGF levels and pathophysiological parameters of schizophrenia. For example, in schizophrenic patients, NGF levels correlated with abnormal electrophysiological parameters, namely p300 potential [34], and antipsychotic treatment was associated with increased serum NGF levels [35]. Therefore, it can be argued that serum NGF levels may be a promising marker of its presence in the CNS. It should be emphasized that NGF dysfunction, caused either by a pathological change in its concentration or by action on the receptor, can contribute to the impairment of neuroplasticity and poor synaptic interactions. These changes may underlie the structural and functional changes seen in schizophrenia. It has been repeatedly reported that serum NGF levels in schizophrenic patients are significantly reduced compared to healthy subjects [36]. It has also been shown that NGF is involved in the regulation and immune functions: it stimulates the proliferation of B and T cells, the production of IgM, IgA, and IgG4 antibodies, chemotaxis, the viability and functional properties of neutrophils, and the survival of memory B cells [30]. It has been found that NGF inhibits the aggressive behavior of male mice and facilitates their subordination by stimulating glucocorticoid secretion in the adrenal cortex [37]. Thus, the high NGF level in paranoid schizophrenia patients with severe negative symptoms may be a mechanism for the development of the symptoms.

In general, it can be suggested that the humoral immunity activation in patients with paranoid schizophrenia occurs due to antigenemia of various origins, and the increased presence of anti-inflammation mediators maintains the activity of type 2 T helpers and, in addition, contributes to latent viral infection. More research is needed to elucidate the causes of immune disorders in schizophrenia with severe negative symptoms, including the genetic factors of their development.

In this work, we aimed to identify changes in brain morphometry that are directly related to the severity of negative symptoms as expressed by neuropsychological data. Confirming this association would not only correlate between the structural and functional changes in the impaired brain but would also explain the differences between phenotypes of paranoid schizophrenia. We have shown that the decrease (p<0.005) in the average cortical thickness in some brain regions (mainly in the frontal lobes) is characteristic of patients with paranoid schizophrenia, regardless of the severity of negative symptoms. This data corroborates the reports showing that schizophrenia is accompanied by structural and functional changes in the brain [38, 39].

In the literature, there are only a few reports about the relationship between negative symptoms and structural changes in the brain. Thus, Zhang et al. [40] found a decrease in the gray matter volume in the cerebellum, the left inferior orbitofrontal cortex, and the right thalamus in schizophrenic patients with negative symptoms. Another work [41] described structural abnormalities (revealed by MRI) in the brain of schizophrenic patients with a neurocognitive deficit: the authors showed a decrease in the volume of the gray and white matter of the brain in this form of schizophrenia. A review by Kaladjian et al. [42] mentioned a decrease in the gray matter density or a decrease in the cortical thickness in the frontal and temporal regions associated with negative symptoms and localized, in particular, in the medial frontal and orbitofrontal cortex, as well as in the area of the amygdala-hippocampal complex.

In this study, we found that the thickness of the cortex in a number of brain regions was reduced (p<0.005) only in patients with high severity of negative symptoms (NSA-4≥15). It should be noted that in these patients, according to morphometric data, there were significant changes in more than 25 parameters (p<0.005 vs control). These results (obtained for the first time) identified a number of regions in both hemispheres where the cortical thickness changed in patients with severe negative symptoms only.

In the present study, we searched for correlations between the immune-inflammatory profile of schizophrenic patients and the structural changes in the brain. No similar studies were found in the available literature. In patients with severe negative symptoms (NSA-4 score≥15), the level of anti-inflammatory cytokine IL-10 negatively correlated with the signal intensity in the basal nuclei and hippocampus (correlation coefficient from –0.74 to –0.42). Moreover, the correlation was strong for the left caudate nucleus, left and right putamen; a moderate correlation was found for the right caudate nucleus, left and right nucleus accumbens, left and right globus pallidus. In total, in patients with high severity of negative symptoms, we found a correlation between the IL-10 level and 60 morphometric parameters. In [26], it has been shown that an increased level of IL-10 is associated with partial disintegration of white matter bundles in schizophrenia. The reasons for these changes are not yet clear, but, in our opinion, they may be due to a high level of systemic neuroinflammation. In this study, we noted similar correlations between the changes in the basal nuclei area and the IL-12p40 cytokine. A number of associations were identified between the IL-12p40 level and brain morphometric parameters; those correlations were characteristic only for schizophrenic patients with severe negative symptoms. Overall, in these patients, correlations between the level of IL-12p40 and more than 73 morphometric indices were found. There was a strong correlation with the folding index in the area of the right frontal pole, a moderate correlation — with the mean curvature in the area of the right transverse temporal gyrus and the right superior temporal gyrus, with the folding index in the area of the left and right sulci, right superior temporal gyrus, right posterior cingulate gyrus, with the area of the cortex in the right posterior cingulate gyrus and the pole of the right temporal lobe, with the mean volume of the cortex in the right posterior cingulate gyrus and in the region of the left amygdala. Notably, in the available literature there is evidence of cytokine penetration across the blood-brain barrier via activated endothelial cells and increased permeability of the blood-brain barrier in schizophrenia [43].

As a result of the study, new information was obtained on the immuno-inflammatory profile and the levels of key regulatory cytokines IL-10 and IL-12p40, as well as neurotrophin NGF in patients with paranoid schizophrenia with severe negative symptoms. We see these results as additional evidence supporting the neuroinflammatory hypothesis of schizophrenia and the role of neuroimmune interactions in the pathogenesis of the disease.

**The limitations of this work** are the relatively small number of patients enrolled in the study, the lack of untreated patients, and the absence of dynamic examination of patients.

## Conclusion

An interdisciplinary approach combining brain morphometry with in-depth immunological and clinical studies of schizophrenic patients, made it possible to identify neurobiological, immune, and neurocognitive markers (IL-10, IL-12p40, NGF), reflecting the relationship between immunological and inflammatory disorders, immunological parameters, and clinical manifestations in schizophrenia. The specifics of immunity disorders and the immune-inflammatory profile of schizophrenic patients with severe negative symptoms were determined. Data were obtained on the close relationship between immune changes and changes in the thickness, gyrification, and cortex volume of the gray matter and ventricles (including important areas of the cortex, hippocampus, and other structures visualized by MRI).

Studies on immunoinflammatory markers of schizophrenia and their correlates in the brain structure are highly important for both science and clinical practice. Considering the variety of forms of schizophrenia and the urgent need for novel therapies, such markers may find their use for monitoring the disease course and assessing the efficacy of novel therapeutic agents.
